# Origination and Immigration Drive Latitudinal Gradients in Marine Functional Diversity

**DOI:** 10.1371/journal.pone.0101494

**Published:** 2014-07-18

**Authors:** Sarah K. Berke, David Jablonski, Andrew Z. Krug, James W. Valentine

**Affiliations:** 1 Dept of Biology, Siena College, Loudonville, New York, United States of America; 2 Dept of the Geophysical Sciences, University of Chicago, Chicago, Illinois, United States of America; 3 Flint Hill School, Oakton, Virginia, United States of America; 4 Department of Integrative Biology, Univ. of California, Berkeley, California, United States of America; Technical University of Denmark, Denmark

## Abstract

Global patterns in the functional attributes of organisms are critical to understanding biodiversity trends and predicting biotic responses to environmental change. In the first global marine analysis, we find a strong decrease in functional richness, but a strong increase in functional evenness, with increasing latitude using intertidal-to-outer-shelf bivalves as a model system (N = 5571 species). These patterns appear to be driven by the interplay between variation in origination rates among functional groups, and latitudinal patterns in origination and range expansion, as documented by the rich fossil record of the group. The data suggest that (i) accumulation of taxa in spatial bins and functional categories has not impeded continued diversification in the tropics, and (ii) extinctions will influence ecosystem function differentially across latitudes.

## Introduction

Understanding global patterns in biodiversity is a fundamental challenge for ecology and evolutionary biology. While nearly two centuries of research have established the ubiquity of latitudinal diversity gradients (LDGs), most analyses have focused on taxonomic diversity. Establishing global patterns in other dimensions of biodiversity, including phylogenetic and functional diversity, is an important prerequisite for understanding the mechanisms driving global patterns and for predicting biotic responses to environmental change [1], [2]. Functional diversity is directly linked to ecosystem function [3], [4], [5], [6], making changes in ecosystem services more likely to result from changes in functional diversity than from species losses *per se*. Determining major patterns in the global deployment of functional diversity is therefore an urgent need in the face of anthropogenic change. Here we present a global analysis of functional diversity patterns in a well-studied marine system, taking advantage of the rich fossil record to illuminate the evolutionary dynamics behind modern patterns.

Our analysis of functional richness and functional evenness focuses on marine Bivalvia, a well-known model system for macroecology and macroevolution. Today, bivalve diversity patterns are representative of the marine biota as a whole [7], [8], and they have a robust fossil record with increasingly well-characterized strengths and biases [8], [9], [10], [11], [12], [13]. We use two standard metrics of functional diversity: functional richness and functional evenness. Functional richness (FR) is the number of functionally unique taxa – we study both species and genera —in a given place and time [14]. Here, functional evenness (FEve) is the extent to which taxa are evenly distributed through the available functional trait space [15], [16], [17]. If clusters of functionally similar taxa exist, then FEve will be low. Conversely, if taxa are spread out to evenly occupy all of the available trait space, then FEve will be high. We expected both FR and FEve to peak in the tropics, the former because of the well-known LDG for bivalves, and the latter because more intense ecological interactions in the taxonomically richest regions might drive taxa to subdivide available niche space more extensively than at higher latitudes [18], [19]. Surprisingly, we found that FR and FEve show discordant trends: FR reaches its maximum in the tropics as expected, but FEve has a strong inverse gradient, with maximum values at high latitudes. This latter pattern is evidently generated by the intersection of two fundamental aspects of bivalve evolution: a tendency for new species and genera to originate in the tropics, and variation in origination rates (perhaps with an ecological filter at high latitudes) among taxonomic groups.

## Methods

### Data Compilation

We defined functional traits for 993 extant bivalve genera living at shelf depths worldwide. Five axes were used, reflecting how bivalves use resources and contribute to ecosystem function: feeding mode; relationship with the substratum; mechanism of attachment; mobility; and body size (Table 1; many sources were used, but valuable compilations include [20], [21], [22]). Feeding mode and body size directly influence energy budgets and reproduction, as well as bivalve contributions to secondary productivity and nutrient cycling. Mobility, attachment, and relationship to the substratum reflect both habitat occupation and the extent to which bivalves physically influence the environment, i.e. through reef formation or bioturbation [23], [24], . Body size — the geometric mean of shell length and height, binned into log_2_ units — correlates well with biomass [26], [27] and with other size metrics [28]. For species- level analyses we used the maximum size reported in the literature, because (a) most species show indeterminate growth [29], (b) the largest size reported for a species correlates well with the largest size sampled from any given population [28], and (c) growth rates slow substantially after maturity, rendering maximum size relatively insensitive to sampling intensity [29]. For genus-level analyses we used the maximum body size in the genus (median or mean size per genus yield similar results, as body size is generally conserved at the genus level). We treat body size as a continuous variable, but binning at different scales did not substantially change the results. Attachment mode and mobility are often not independent, but some byssate forms are mobile just as some unattached forms are immobile, making the distinction necessary. Similarly, swimming is a special case of mobility that we treat separately because it confers a capacity for dispersal and predator avoidance that goes beyond the simple capacity to change position or re-burrow after disturbance.

**Table 1:** Functional axes used in this study.

*Category*	*Levels*
Feeding mode	suspension feeding
	surface deposit-feeding
	subsurface deposit-feeding
	photosymbiosis
	chemosymbiosis
	carnivory
Substrate relationships	epifaunal
	infaunal siphonate
	infaunal asiphonate
	semi-infaunal
	nestling
	boring
Attachment mode	unattached
	byssate
	cemented
Mobility	immobile
	mobile
	swimming
Body Size	log_2_(√(shell length · height))

These data were cross-tabulated with a global database of >50,000 individual species occurrences for 5571 species from 3442 shelf-depth localities worldwide (Figure S1, date of download March 20 2012). Localities vary in spatial resolution from points (e.g. Horn Island, Australia) to larger regions (e.g. New South Wales, Australia), reflecting uneven sampling and reporting across the world oceans.

### Functional Diversity Metrics and Null Models

Functional richness (FR) and functional evenness (FEve) were calculated for 10° latitudinal bands along the shelves and shallow island platforms of each of the 4 major N-S coastlines: the West Atlantic, the East Atlantic, the West Pacific, and the East Pacific. The longitudinal extent of each band was determined by the localities in which occurrence data exist. In general, longitudinal bands were within a few degrees of the coastline, the exception being oceanic islands (especially in the West Pacific). For bivalves, island faunas are essentially attenuated subsets of the nearest mainland faunas (Jablonski unpublished data), with some allopatric replacements of mainland species by related island endemics at the most distant islands, and so we allow those localities to fall within the longitudinal extent of each bin. We next applied a standard range-through assumption: species not occurring in a given band, but occurring both to the north and south of it, were assumed to occur in that band (relaxing this assumption yields similar results). All analyses were conducted at both the genus and species level. Results were similar, and only genus-level analyses are shown here, to simplify integration with fossil data (see Figure S2 for comparison to species-level patterns). We also investigated Functional Divergence, but found few robust patterns; methods and data are presented in (Text S1) and Figure S3.

#### Functional richness

FR was defined as the number of unique trait combinations observed in each 10° band along each coastline [14], [15], [30], using the functional categories described above. Body size was excluded from this analysis to avoid problems associated with binning a continuous variable; preliminary analyses including body size with various binning schemes yielded similar results.

#### Functional evenness

FEve is a distance-based metric based on principal coordinates analysis (PCoA) [15] (see also [16], [17]). For each of the four coastlines, a PCoA was conducted for all genera occurring on that coastline. FEve was then calculated from the branch lengths of the minimum spanning tree for the subset of genera occurring in each latitudinal bin (using the R package ‘FD’, [31], [32]). FEve is independent of taxonomic richness when traits are distributed randomly among taxa [15]. High values of FEve indicate that genera are evenly distributed throughout the available trait space; low values of FEve indicate an uneven or clumped distribution, with some regions of trait space more heavily occupied than others.

#### Null modeling

A null expectation for FR was generated by randomly drawing genera from the pool of all genera on each coastline, matching the observed genus richness in each latitudinal bin. For FEve, we randomly drew PCoA coordinates from the pool of all genera on each coastline, again matching the observed genus richness for each latitudinal bin. For both FR and FEve, we conducted 1000 iterations for each latitudinal bin on each coastline. We then compared the slopes of FR and FEve versus latitude for the null predictions to the observed data using Gaussian linear models. Observations were considered significantly different from the null if they fell in the upper or lower 2.5% of the null distribution, consistent with alpha  = 0.05.

### Origination rates

Origination rates (in genera per million years [my]) were estimated from backwards survivorship curves based on paleontological data for the distribution of genus ages – i.e. the first occurrence of each genus in the fossil record — within each functional group [33], [34]. Body size was excluded from functional groupings in these analyses because even coarse bins rendered most groups with too few genera to analyze. Some functional categories were combined to obtain a sample size that would yield well-supported estimates of origination rate (Table 2).

**Table 2:** Origination rates for bivalve functional groups.

Functional Group	Rate	N	AIC
suspension, epifaunal, immobile, cemented	0.030	45	406.2
suspension, infaunal asiphonate, mobile, unattached	0.033	70	619.1
suspension, infaunal siphonate, immobile, unattached	0.015	6	64.4
subsurface deposit, infaunal asiphonate, mobile, unattached	0.018	13	132.1
chemosymbiotic, infaunal siphonate, mobile, unattached	0.023	60	573.5
subsurface deposit, infaunal siphonate, mobile, unattached	0.019	19	190.1
surface deposit, infaunal siphonate, mobile, unattached	0.030	54	488.8
suspension, infaunal siphonate, mobile, unattached	0.042	325	2719.8
all carnivores	0.034	35	307.9
all photosymbionts	0.065	6	46.9
all borers	0.022	38	368.8
all swimmers	0.015	11	115.7
all byssate	0.020	115	1128.9

Origination rates (in genera per million years) for each functional group, as estimated from backwards survivorship curves [33], [34]. N indicates the number of genera in each group; it was necessary to combine some groups to achieve adequate sample sizes. AIC indicates the degree of support for the rate estimate.

## Results and Discussion

**Figure 1:**
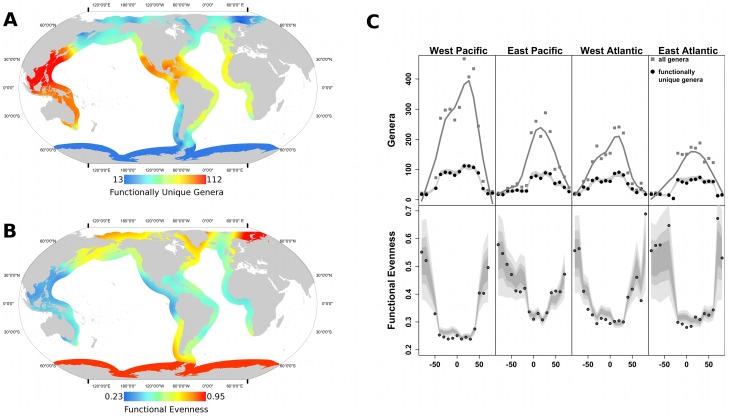
Global patterns in Functional richness (FR) and Functional evenness (FEve) for marine bivalves. Heatmaps are interpolated from data for 10° latitudinal bands for (A) FR and (B) FEve. (C) Raw observations are plotted against latitude, showing the LDG in taxonomic richness (top, gray squares and black line), functional richness (top, black circles and gray line), and functional evenness (bottom). For both plots, the region in pale gray represents the central 95% of FRs or FEves from 1000 random draws for each latitude/coast combination. Observed FR and FEve are both broadly consistent with the null expectations given the taxonomic richness of each bin, and thus consistent with the high-latitude fauna being an attenuated sample of the global genus pool.

We found strong latitudinal gradients in both functional richness and functional evenness for all coastlines at both the genus and species levels (Figure 1, Figures S2, S4, S5). As expected, functional richness exhibits a low-latitude peak, and a saturating relationship with genus richness (as expected given that the number of taxa exceeds the number of possible functional groups). In our categorization scheme, 197 functional combinations seem biologically possible, yet only 39 occur in our data. For example, many infaunal suspension feeders attach bysally to the sediment; it therefore seems plausible that infaunal deposit feeders could also be byssate, yet there are no byssate infaunal deposit-feeders in our data. (In contrast, it would be plainly impossible for any bivalve to be simultaneously cemented to the substratum yet also mobile). Of the 158 combinations that seem plausible yet do not exist in our data, 58% involve the rarest feeding modes — carnivory, photosymbiosis, and chemosymbiosis — while a further 35% involve deposit feeding. These patterns emphasize that most living bivalves are suspension feeders, and other trophic modes have not diversified as extensively.

We expected that FEve would also be maximized at low latitude, given the high taxonomic diversity and theoretically larger number of niches in the tropics, and the potential for competition to cause saturation within functional categories. Instead, FEve exhibits a strong inverse latitudinal gradient, with lowest values in the tropics. This finding is consistent with global data for spiders [35] and New World bats [36], but inconsistent with other data for bats [1] and for woody plants, which show variable results depending on the evenness metric used [2]. Marine fishes show similar global diversity patterns [37], but analyses have emphasized community evenness rather than functional evenness [38], and so are not directly comparable to our results.

**Figure 2:**
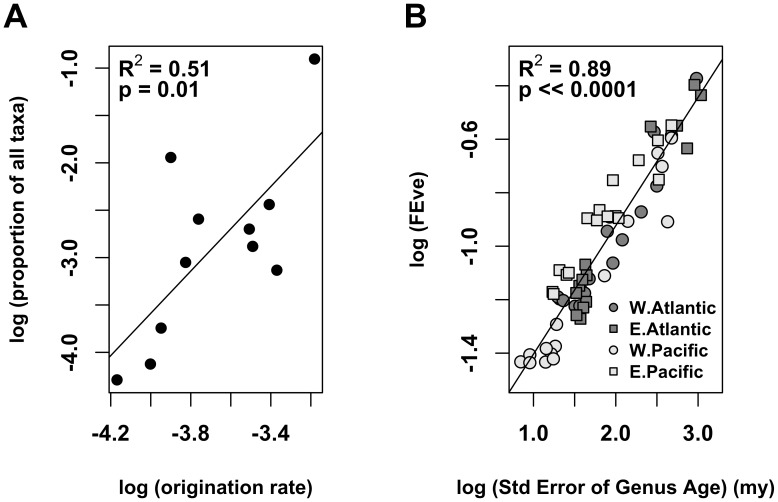
Origination rates for bivalve functional groups. Origination rates (genera · my^−1^) vary among bivalve functional groups. As a result, groups with higher origination rates represent a larger proportion of the overall fauna, contributing to the inherent unevenness in the bivalve gene pool (A, each point represents a distinct functional group, see Table 2). When coupled with an “Out of the Tropics” dynamic, FEve and the standard error in genus age both correlate strongly with latitude, and with each other (B). Each point represents a single 10° band on a given coastline; regressions were calculated for all coastlines pooled together. Origination rates are based on geological age-frequency distributions of extant genera in the form of “backwards survivorship curves” [33], [34].

We hypothesized that the latitudinal pattern in FEve arises through the interaction of two evolutionary patterns: First, bivalve origination rates vary with functional group (Table 2, Figure 2). Those functional groups that generate new taxa more rapidly come to represent a larger proportion of the bivalve fauna (Figure 2A), creating an inherently uneven genus pool (FEve 0.35-to 0.44 for the pool of genera on each coastline). Second, the fossil record provides strong evidence that bivalve genera disproportionately arise in the tropics and then expand to higher latitude [8], [39]. This ‘Out of the Tropics’ (OTT) dynamic creates a gradient in genus age — with variance decreasing with latitude mainly by a decrease in the proportion of young genera — that correlates with the observed gradient in FEve (Figure 2B and [39]).

**Figure 3:**
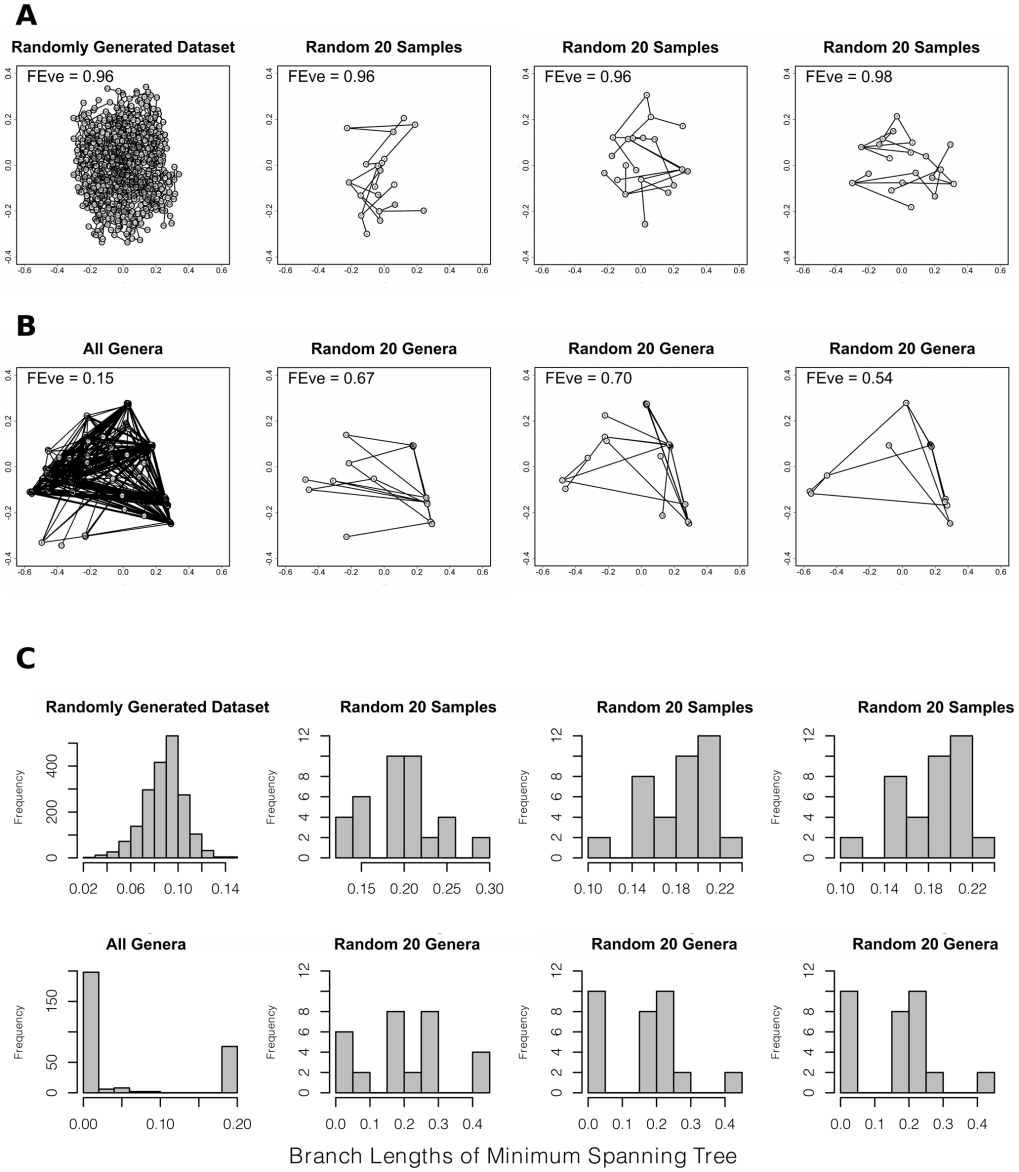
Random sampling of even and uneven taxon pools. FEve is calculated from branch lengths of the minimum spanning tree (MST) connecting all taxa in a functional ordination [15]. When a taxon pool with low FEve is randomly sampled, the samples have higher FEve, as illustrated here. Panels A and B are PCoA plots with MSTs superimposed. For both panels, the left-most plot shows a taxon pool while the three right-most plots show three random samples from that pool. Panel A shows a randomly generated dataset, having the same dimensions as the bivalve genus data — a very even taxon pool. Randomly sampling from an even pool does not alter the evenness. Panel B shows data for all bivalve genera — an uneven taxon pool. Randomly sampling from an uneven taxon pool causes FEve to increase. (The pattern holds for 1000 random samples, as shown in Figure 1C). Panel C shows the distribution of MST branch lengths for each of the 8 plots in panels A-B. Despite the highly skewed distribution of branch lengths for the pool of all genera, samples from that pool have a relatively more even distribution of branch lengths than seen in the entire pool.

Taken together, these patterns can explain the observed gradient in FEve. Because functional groups have different origination rates, and because genera disproportionately arise in the tropics, the tropical genus pool is functionally uneven. Because extratropical faunas are seeded from the tropics, they represent a subsample of the uneven genus pool. Small samples of an uneven pool capture that unevenness imperfectly, and therefore have higher evenness (Figure 3). Consequently, high-latitude bivalve faunas are more even than their tropical counterparts. The null model results shown in Figure 1 support this view by showing that 1000 random draws from an uneven genus pool are generally higher in FEve than the underlying pool. The point is further illustrated in Figure 3, which shows minimum spanning trees and the distributions of branch lengths for those trees when sub-samples are taken from even versus uneven taxon pools.

To further test the OTT aspect of our hypothesis, we repeated our null modeling, this time drawing only from the pool of tropical genera, defined as those genera occurring between 20°N and 20°S latitude. The results were indistinguishable from the results for all genera (Figure 1), because so few genera are exclusively extratropical that the tropical genus pool is virtually identical to the total genus pool for each coast. Both models generate the observed pattern with high accuracy. FEve does fall below the null expectation in the tropical West Pacific, southern West Atlantic, and southern East Atlantic. This serves to emphasize the extreme unevenness of the bivalve genus pool, which is diminished by subsampling even when a large proportion of the starting pool is sampled. Overall, we conclude that the concordance between the null predictions and observed FEve is consistent with an OTT world in which new taxa arise disproportionately among functional groups. Previous work has found FEve to be independent of taxon richness [15], but our analysis emphasizes that the metrics are only independent if traits are randomly distributed among taxa. When FEve is low in the underlying taxon pool, then FEve will be inversely related to richness (Figures 3 and S6)

Because origination rates vary among functional groups and also peak in the tropics, bivalves exhibit heightened functional redundancy in the tropics (Figures S5, S7, Table S1). Indeed, as many as 145 genera occupy a single functional bin in the tropical West Pacific, and tropical bins average 5.8±12.9 genera per functional group compared to 1.0±3.0 at boreal latitudes (mean ± SD, Figure 1, Figure S7, Table S1). Presumably, the bivalve species are partitioning the niche space more finely than is captured by our coarse-grained groupings — an hypothesis that would make interesting future work (although unlikely to change our results, because finer distinctions would only slightly offset species within the ordinations constructed here). In contrast to woody plants, we see little evidence for selective filtering of functional traits at higher latitudes. The functional groups that drop out at high latitude exhibit few unifying characteristics. Only photosymbiotic, boring, and infaunal byssate forms are entirely absent at high latitude for both poles on all 4 coastlines (Figure S7). Photosymbionts clearly require intense solar irradiance to function, preventing their occurrence at high latitude. Borers require wood debris or soft rock, and wood at least may be limiting at polar latitudes. Borers also tend to be shallow-water species, and may thus be limited by seasonal ice. Infaunal byssate forms are uncommon at any latitude, and their absence near the poles may be a vagary of low richness, or may reflect spatial variation in selective pressures, perhaps owing to different suites of predators or to different energetic constraints in cold waters.

More subtle shifts in evenness may also be environmentally driven. For example, the tropics-to-Arctic shift of the ratio of burrowing carnivores to burrowing siphonate suspension feeders, from 10/145 to 2/3, probably reflects the difficulties of phytoplankton feeding in the severe and highly seasonal polar environment, but also increases evenness as the suspension feeders decline in relative diversity. Similarly, some other smaller groups (e.g. certain deposit-feeders, perhaps owing to a mode of larval development apparently favored at high latitudes) have near-constant or even increasing diversities when traced into high latitudes [40]. Such patterns emphasize that, although FEve is broadly consistent with the null model presented here, biological factors undoubtedly contribute to functional diversity patterns at global scales. These likely effects will require additional data to separate from the first-order mechanism outlined here.

## Conclusion

Our results show that the latitudinal diversity gradient is characterized not only by changes in taxon richness, but by changes in the occupation of functional space shaped in part by large-scale evolutionary and biogeographic dynamics of the clade as a whole. Functional richness follows a predictable saturating function of taxonomic richness. The low evenness/high redundancy observed in the tropics evidently arises from (i) differential origination rates among functional groups, and (ii) an ‘Out of the Tropics’ dynamic underlying bivalve origination and range expansion [8], [39]. At large scales, the occupation of functional space appears to be driven primarily by evolutionary dynamics, as functional unevenness is no more attenuated at high latitudes than expected for small-sample draws from the tropical (or global) genus pool, consistent with the OTT hypothesis. In general, functional groups persist across latitudes despite dramatic decreases in taxonomic diversity. This suggests that the observed functional combinations represent evolutionarily stable strategies for bivalves, which are robust to dramatically variable environmental conditions. It would be interesting to know whether other lineages exhibit similar stability in functional guilds. While a few functional groups are evidently excluded from high latitudes for ecological reasons, they represent a small proportion of the total bivalve fauna and thus do not drive departures from the null expectation. In contrast to woody plants [2], our results imply a “supply-side” model for this marine system, in which external constraints or saturation effects are secondary in the tropics, and the occupation of functional space is determined primarily by the differential origination rates among functional groups, which is most pronounced in the tropics because overall origination rates are higher there. Together with the lack of evidence for a recent downturn in origination rates for these functional groups (ms in preparation), these data suggest that not all niches are occupied and the tropics are not full despite the enormous diversity they contain.

Lower redundancy at high latitudes suggests that the impact of past and future extinction on ecosystem function will vary with latitude. For a given extinction intensity, high-latitude extinctions may yield larger shifts in ecosystem function than lower-latitude events. Of course, species' ecosystem-level effects can vary dramatically even within functional groups, and species in a single functional group (as we define them) may be ecologically different in myriad more subtle ways [41]. Nevertheless, models of anthropogenic species decline or loss should take functional roles into account, and should evaluate whether the inverse gradient in FEve is a widespread phenomenon. Our data suggest that major groups with uneven overall distributions of taxa among functional groups, as in bivalves, should show lowest FEve in areas of highest taxonomic richness, while groups that fill the available functional space more evenly may damp or lack latitudinal FEve patterns. Given the inextricable link between functional diversity and ecosystem function, meeting the challenges of environmental change requires a fuller understanding of how functional diversity is deployed globally, and of the ecological and evolutionary mechanisms driving these patterns.

## Supporting Information

Figure S1 **Localities used for each coastline.** Squares  =  Western Pacific, diamonds  =  Eastern Pacific, triangles  =  Western Atlantic, circles  =  Eastern Atlantic. World Hammer-Aitoff projection.
(TIF)

Figure S2 **FEve shows similar patterns whether evaluated at the species level or at the genus level. [Sarah, should this be rotated 90 degrees, to be consistent with** **Fig. 1** **in the main text?**
(EPS)

Figure S3 **Functional divergence (FDiv) for each coastline.** Black symbols indicate the observed pattern for extant genera, white squares indicate the pattern with extinct genera added to each of the two polar bins (see [42]). Adding extinct Arctic genera partially resolves the hemispheric asymmetry for the Pacific basin, but has the opposite effect or no effect in the West and East Atlantic, respectively.
(EPS)

Figure S4 **Observed versus expected slopes for FR and FEve with latitude.** The observed relationships between (i) functional richness (FR) and latitude and (ii) functional evenness (FEve) and latitude are generally consistent with expectations from a null model based on randomly assembling communities from the coast-wide genus pool. Gaussian linear models were constructed for FR or FEve vs. latitude for each hemisphere of each coastline (because there is no *a priori* reason to assume that patterns will be symmetrical about the equator for any given coastline). Histograms of slopes from 1000 model runs are shown in gray; the observed slopes are shown as a black vertical line. The 2.5^th^ and 97.5^th^ quantiles of the null distributions are shown as vertical dotted lines. Departures from the null model were considered significant if they fell within the outermost 5% of the distribution, as indicated by asterisks in the upper left hand corner. Only 2 coastlines/hemispheres were significant, but the directions differed, with one observation being steeper than expected and the other two being less steep (note that the observed slope for the southern East Atlantic is −565, beyond the range of the plot). Thus, it is difficult to ascribe biological meaning to these departures.
(EPS)

Figure S5 **Principal coordinates plots showing the first two PCoA axes for each latitudinal bin on each coastline.** FEve was calculated based on the minimum spanning tree for each set of PCoA axes. Each point represents a genus; increased functional redundancy at low latitude is reflected by tight clusters of genera in functional space.
(TIF)

Figure S6 **Functional evenness versus richness.** A: Data for marine bivalves. Functional evenness (FEve) exhibits a negative relationship with both taxon (genus) richness and functional richness (number of functionally unique genera). B: Data from Villeger et al. [15]. When traits are randomly distributed among taxa, there is no relationship between FEve and taxon richness or functional richness (FRic) (plots in panel B are modified from Villeger et al. Figure 4 [15]).
(TIF)

Figure S7 **Mosaic plots showing the distribution of functional groups for each coastline.** Five separate files are provided: the figure key, and figure files S7A-D. Figure S7 Key. Figure S7A: Western Pacific. Figure S7B: Eastern Pacific. Figure S7C: Western Atlantic. Figure S7D: Eastern Atlantic.The width of each box indicates the relative richness of the latitudinal band; the height indicates the relative abundance of each functional group within the latitudinal band. Borers, infaunal byssate genera, and photosymbiotic genera are highlighted in gray to emphasize that they are the only groups that are present at low latitude but consistently absent at high latitude. Dashed lines indicate that no taxa falling into that category were recorded at shelf depths for the corresponding latitudinal bin. The numbers of genera in each cell are shown in Table S1. Note that these mosaics (and Table S1) do not include body size data, because doing so would generate more groups than can be visually represented. These data are therefore not directly comparable to the data from Figure 2. Functional groups are color coded as shown in the figure key.
(TIFF)

Table S1 **The number of genera occupying each unique functional group for each coastline/latitude.** The mean and maximum number of genera per functional group decreases from low to high latitude (see Figure S7 key). Body size was not included in these functional groups.
(DOC)

Text S1 **Supplementary methods.**
(PDF)
