# Supplementary figures and images for: Origination and Immigration Drive Latitudinal Gradients in Marine Functional Diversity
Source: PLoS One. 2014 Jul 18;9(7):e101494. doi: 10.1371/journal.pone.0101494 (PMC4103801; doi:10.1371/journal.pone.0101494)

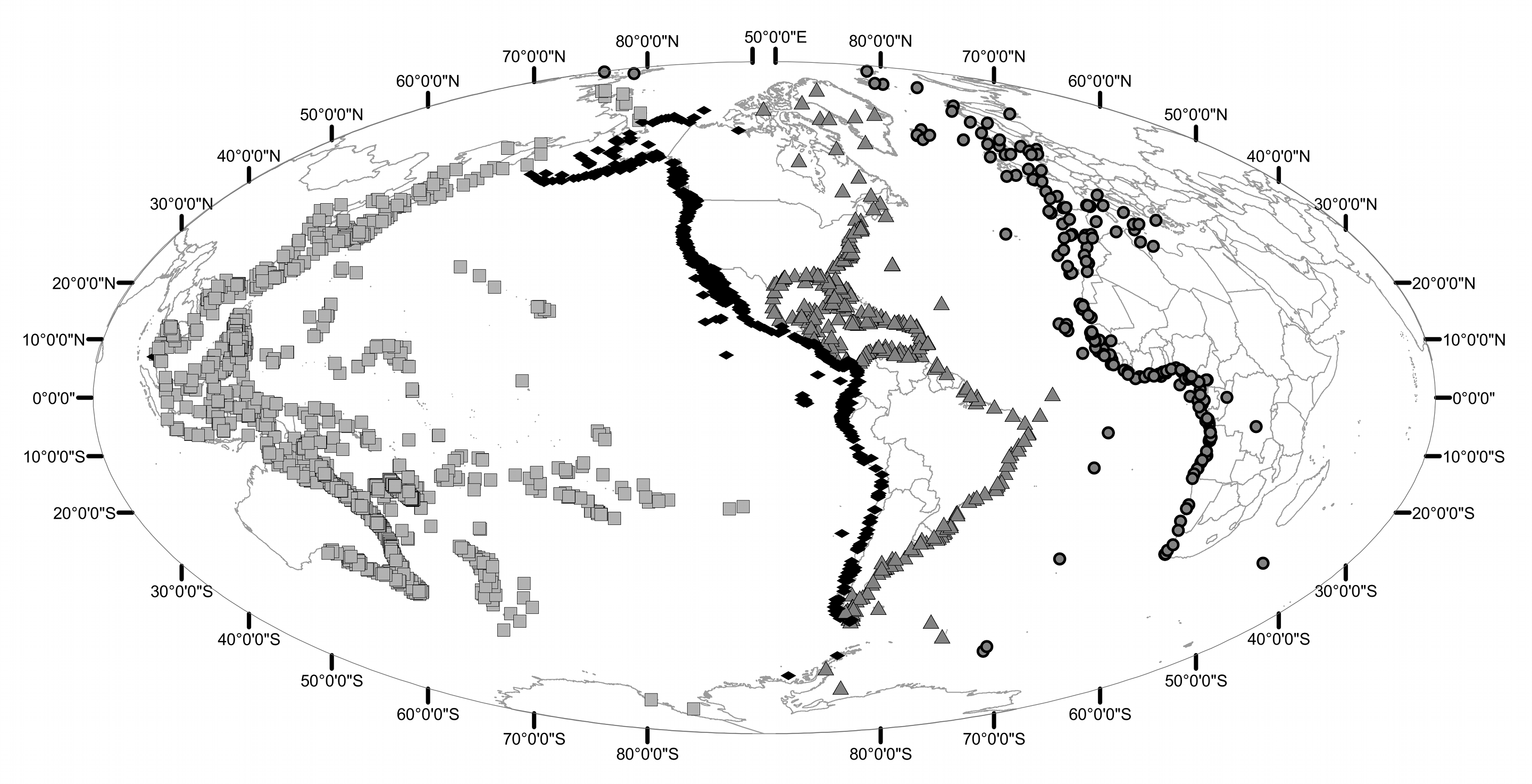

Supplement: Figure S1 — Localities used for each coastline. Squares = Western Pacific, diamonds = Eastern Pacific, triangles = Western Atlantic, circles = Eastern Atlantic. World Hammer-Aitoff projection. (TIF) [file pone.0101494.s001.tif]

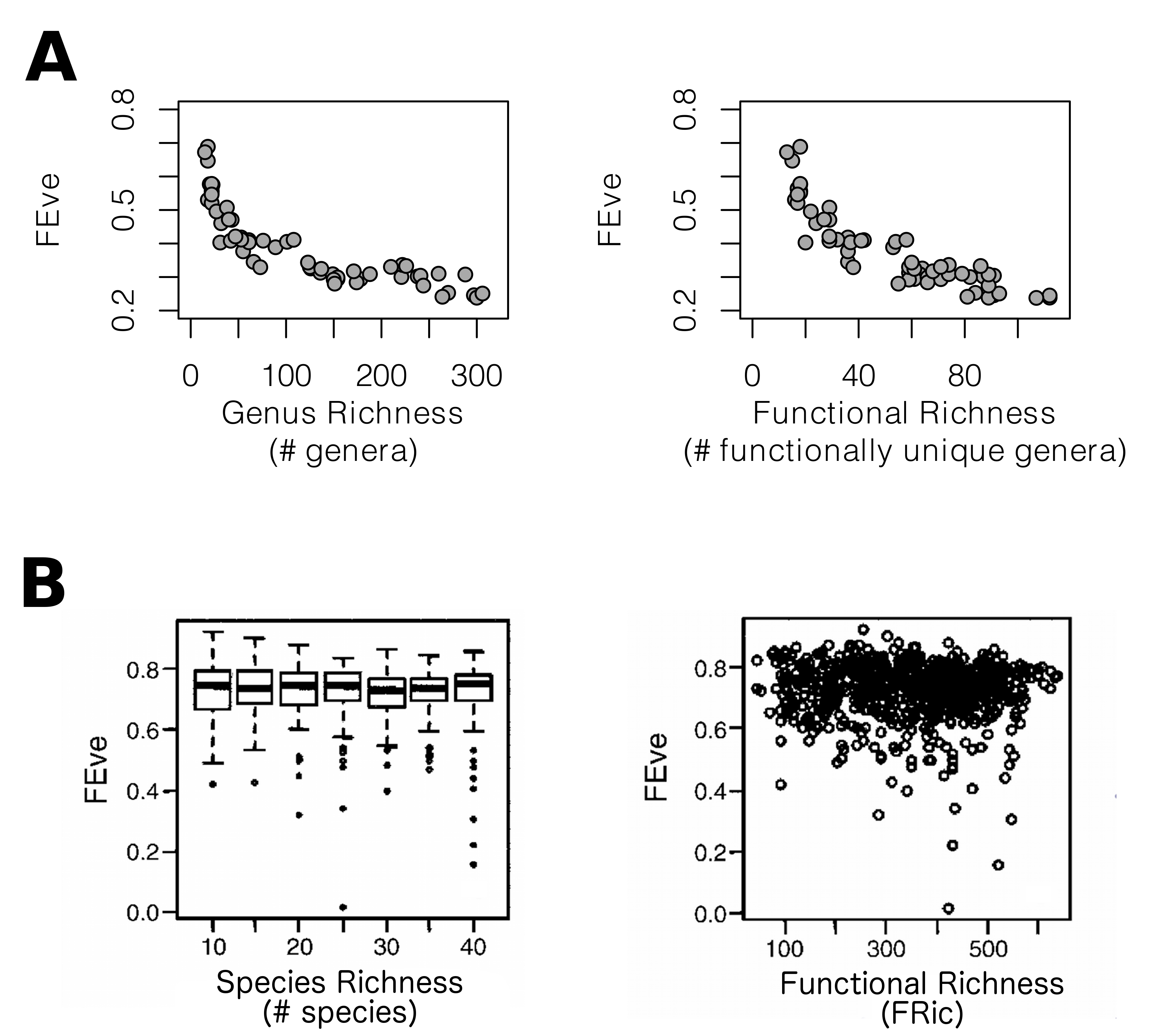

Supplement: Figure S6 — Functional evenness versus richness. A: Data for marine bivalves. Functional evenness (FEve) exhibits a negative relationship with both taxon (genus) richness and functional richness (number of functionally unique genera). B: Data from Villeger et al. [15]. When traits are randomly distributed among taxa, there is no relationship between FEve and taxon richness or functional richness (FRic) (plots in panel B are modified from Villeger et al. Figure 4 [15]). (TIF) [file pone.0101494.s006.tif]
