# Supplementary material for: Origination and Immigration Drive Latitudinal Gradients in Marine Functional Diversity
Source: PLoS One. 2014 Jul 18;9(7):e101494. doi: 10.1371/journal.pone.0101494 (PMC4103801; doi:10.1371/journal.pone.0101494)
Supplement: Table S1 — The number of genera occupying each unique functional group for each coastline/latitude. The mean and maximum number of genera per functional group decreases from low to high latitude (see Figure S7 key). Body size was not included in these functional groups. (DOC) [file pone.0101494.s008.doc]

**Table S1: The number of genera occupying each unique functional group for each coastline / latitude.**

| **West Atlantic** | **-80** | **-70** | **-60** | **-50** | **-40** | **-30** | **-20** | **-10** | **0** | **10** | **20** | **30** | **40** | **50** | **60** | **70** | **80** |
| --- | --- | --- | --- | --- | --- | --- | --- | --- | --- | --- | --- | --- | --- | --- | --- | --- | --- |
| carnivore / epifaunal / byssate / mobile | 1 | 1 | 2 | 0 | 1 | 1 | 1 | 1 | 1 | 1 | 2 | 1 | 1 | 1 | 1 | 2 | 2 |
| carnivore / epifaunal / byssate / swimming | 0 | 0 | 0 | 0 | 0 | 1 | 1 | 0 | 0 | 1 | 1 | 1 | 1 | 0 | 0 | 0 | 0 |
| carnivore / infaunal_siphonate / unattached / mobile | 2 | 2 | 3 | 0 | 6 | 7 | 4 | 4 | 4 | 8 | 9 | 6 | 5 | 2 | 1 | 4 | 2 |
| chemosymbiotic / infaunal_asiphonate / unattached / mobile | 0 | 0 | 0 | 0 | 0 | 0 | 1 | 1 | 0 | 0 | 0 | 0 | 0 | 0 | 0 | 0 | 0 |
| chemosymbiotic / infaunal_siphonate / unattached / mobile | 2 | 3 | 6 | 2 | 5 | 11 | 4 | 7 | 14 | 19 | 20 | 19 | 7 | 2 | 1 | 6 | 0 |
| chemosymbiotic / infaunal_siphonate / unattached / swimming | 0 | 0 | 0 | 0 | 0 | 0 | 0 | 0 | 0 | 0 | 0 | 0 | 0 | 0 | 0 | 0 | 0 |
| photosymbiotic / epifaunal / byssate / immobile | 0 | 0 | 0 | 0 | 0 | 0 | 0 | 0 | 0 | 0 | 0 | 0 | 0 | 0 | 0 | 0 | 0 |
| photosymbiotic / epifaunal / unattached / mobile | 0 | 0 | 0 | 0 | 0 | 0 | 0 | 0 | 0 | 0 | 0 | 0 | 0 | 0 | 0 | 0 | 0 |
| photosymbiotic / semi_infaunal / unattached / mobile | 0 | 0 | 0 | 0 | 0 | 0 | 0 | 0 | 0 | 0 | 0 | 0 | 0 | 0 | 0 | 0 | 0 |
| subsurface_deposit / infaunal_asiphonate / unattached / mobile | 2 | 1 | 3 | 2 | 2 | 2 | 2 | 2 | 1 | 4 | 4 | 3 | 2 | 1 | 1 | 1 | 1 |
| subsurface_deposit / infaunal_siphonate / unattached / immobile | 0 | 0 | 0 | 0 | 0 | 0 | 0 | 0 | 0 | 1 | 0 | 0 | 0 | 0 | 0 | 0 | 0 |
| subsurface_deposit / infaunal_siphonate / unattached / mobile | 2 | 2 | 3 | 3 | 5 | 6 | 2 | 3 | 2 | 3 | 3 | 6 | 3 | 4 | 3 | 4 | 2 |
| surface_deposit / infaunal_siphonate / unattached / mobile | 1 | 1 | 2 | 6 | 15 | 22 | 22 | 23 | 24 | 34 | 31 | 27 | 7 | 3 | 2 | 2 | 2 |
| suspension / borer / byssate / immobile | 0 | 0 | 1 | 1 | 1 | 2 | 0 | 2 | 1 | 4 | 3 | 3 | 0 | 0 | 0 | 0 | 0 |
| suspension / borer / cemented / immobile | 0 | 0 | 0 | 0 | 0 | 0 | 0 | 0 | 0 | 0 | 0 | 0 | 0 | 0 | 0 | 0 | 0 |
| suspension / borer / unattached / immobile | 0 | 0 | 2 | 4 | 8 | 7 | 6 | 9 | 9 | 10 | 12 | 13 | 4 | 1 | 0 | 0 | 0 |
| suspension / borer / unattached / mobile | 0 | 0 | 0 | 2 | 3 | 4 | 3 | 2 | 3 | 5 | 5 | 4 | 1 | 0 | 0 | 0 | 0 |
| suspension / epifaunal / byssate / immobile | 0 | 0 | 7 | 3 | 10 | 16 | 13 | 14 | 12 | 19 | 21 | 20 | 7 | 6 | 5 | 6 | 1 |
| suspension / epifaunal / byssate / mobile | 4 | 4 | 7 | 7 | 9 | 8 | 5 | 6 | 7 | 10 | 11 | 7 | 2 | 1 | 1 | 2 | 1 |
| suspension / epifaunal / byssate / swimming | 2 | 2 | 3 | 1 | 3 | 4 | 1 | 4 | 4 | 4 | 5 | 7 | 5 | 4 | 1 | 1 | 1 |
| suspension / epifaunal / cemented / immobile | 0 | 0 | 1 | 4 | 7 | 10 | 10 | 9 | 7 | 17 | 16 | 15 | 3 | 1 | 0 | 1 | 0 |
| suspension / epifaunal / unattached / immobile | 0 | 0 | 0 | 0 | 1 | 2 | 1 | 2 | 2 | 2 | 2 | 2 | 1 | 0 | 0 | 0 | 0 |
| suspension / epifaunal / unattached / mobile | 0 | 0 | 0 | 0 | 0 | 0 | 0 | 1 | 1 | 1 | 1 | 0 | 0 | 0 | 0 | 0 | 0 |
| suspension / epifaunal / unattached / swimming | 1 | 1 | 1 | 0 | 0 | 0 | 0 | 0 | 0 | 0 | 0 | 0 | 0 | 0 | 0 | 0 | 0 |
| suspension / infaunal_asiphonate / byssate / immobile | 0 | 0 | 0 | 0 | 0 | 0 | 0 | 0 | 0 | 0 | 0 | 0 | 0 | 0 | 0 | 0 | 0 |
| suspension / infaunal_asiphonate / byssate / mobile | 0 | 0 | 0 | 0 | 2 | 2 | 2 | 2 | 2 | 3 | 2 | 1 | 0 | 0 | 0 | 0 | 0 |
| suspension / infaunal_asiphonate / unattached / immobile | 0 | 0 | 0 | 0 | 1 | 2 | 2 | 2 | 2 | 5 | 5 | 5 | 1 | 1 | 1 | 1 | 1 |
| suspension / infaunal_asiphonate / unattached / mobile | 1 | 1 | 3 | 3 | 5 | 6 | 4 | 7 | 6 | 11 | 11 | 11 | 4 | 2 | 2 | 4 | 2 |
| suspension / infaunal_siphonate / byssate / immobile | 0 | 0 | 0 | 3 | 3 | 5 | 3 | 2 | 1 | 3 | 3 | 2 | 1 | 0 | 0 | 0 | 0 |
| suspension / infaunal_siphonate / byssate / mobile | 0 | 0 | 0 | 0 | 0 | 0 | 0 | 0 | 0 | 0 | 0 | 0 | 0 | 0 | 0 | 0 | 0 |
| suspension / infaunal_siphonate / unattached / immobile | 0 | 0 | 0 | 1 | 1 | 1 | 0 | 0 | 0 | 0 | 1 | 2 | 1 | 1 | 0 | 1 | 0 |
| suspension / infaunal_siphonate / unattached / mobile | 3 | 3 | 15 | 22 | 32 | 49 | 42 | 39 | 41 | 58 | 59 | 52 | 29 | 20 | 11 | 18 | 2 |
| suspension / infaunal_siphonate / unattached / swimming | 0 | 0 | 0 | 0 | 0 | 1 | 1 | 1 | 1 | 1 | 1 | 1 | 1 | 0 | 0 | 0 | 0 |
| suspension / nestler / byssate / immobile | 0 | 0 | 1 | 1 | 2 | 3 | 2 | 3 | 4 | 5 | 4 | 4 | 1 | 1 | 1 | 1 | 1 |
| suspension / nestler / byssate / mobile | 0 | 0 | 0 | 0 | 0 | 1 | 1 | 1 | 1 | 2 | 2 | 1 | 0 | 0 | 0 | 0 | 0 |
| suspension / semi_infaunal / byssate / immobile | 0 | 0 | 0 | 1 | 1 | 2 | 1 | 1 | 1 | 4 | 4 | 4 | 1 | 1 | 0 | 0 | 0 |
| suspension / semi_infaunal / unattached / immobile | 0 | 0 | 0 | 0 | 0 | 0 | 0 | 0 | 0 | 0 | 0 | 0 | 0 | 0 | 0 | 0 | 0 |
| suspension / semi_infaunal / unattached / mobile | 1 | 1 | 1 | 0 | 3 | 3 | 2 | 1 | 3 | 3 | 3 | 4 | 1 | 1 | 1 | 1 | 0 |
| suspension / semi_infaunal / unattached / swimming | 0 | 0 | 0 | 0 | 0 | 0 | 0 | 0 | 0 | 0 | 0 | 0 | 0 | 0 | 0 | 0 | 0 |
| **Mean** | **0.5** | **0.5** | **1.5** | **1.6** | **3.2** | **4.5** | **3.4** | **3.8** | **3.9** | **6.1** | **6.1** | **5.6** | **2.2** | **1.3** | **0.8** | **1.4** | **0.4** |
| **Max** | **4** | **4** | **15** | **22** | **32** | **49** | **42** | **39** | **41** | **58** | **59** | **52** | **29** | **20** | **11** | **18** | **2** |

| **East Atlantic** | **-80** | **-70** | **-60** | **-50** | **-40** | **-30** | **-20** | **-10** | **0** | **10** | **20** | **30** | **40** | **50** | **60** | **70** | **80** |
| --- | --- | --- | --- | --- | --- | --- | --- | --- | --- | --- | --- | --- | --- | --- | --- | --- | --- |
| carnivore / epifaunal / byssate / mobile | 1 | 1 | 1 | 0 | 1 | 0 | 1 | 0 | 1 | 2 | 3 | 3 | 1 | 2 | 2 | 1 | 2 |
| carnivore / epifaunal / byssate / swimming | 0 | 0 | 0 | 0 | 0 | 0 | 0 | 0 | 0 | 0 | 0 | 0 | 0 | 0 | 0 | 0 | 0 |
| carnivore / infaunal_siphonate / unattached / mobile | 2 | 2 | 2 | 0 | 1 | 0 | 3 | 2 | 3 | 1 | 5 | 7 | 3 | 6 | 5 | 0 | 0 |
| chemosymbiotic / infaunal_asiphonate / unattached / mobile | 0 | 0 | 0 | 0 | 0 | 0 | 0 | 0 | 0 | 0 | 0 | 0 | 0 | 0 | 0 | 0 | 0 |
| chemosymbiotic / infaunal_siphonate / unattached / mobile | 2 | 3 | 2 | 0 | 0 | 0 | 5 | 9 | 19 | 10 | 11 | 13 | 8 | 12 | 10 | 0 | 0 |
| chemosymbiotic / infaunal_siphonate / unattached / swimming | 0 | 0 | 0 | 0 | 1 | 0 | 1 | 1 | 0 | 1 | 1 | 1 | 0 | 0 | 0 | 0 | 0 |
| photosymbiotic / epifaunal / byssate / immobile | 0 | 0 | 0 | 0 | 0 | 0 | 0 | 0 | 0 | 0 | 0 | 0 | 0 | 0 | 0 | 0 | 0 |
| photosymbiotic / epifaunal / unattached / mobile | 0 | 0 | 0 | 0 | 0 | 0 | 0 | 0 | 0 | 0 | 0 | 0 | 0 | 0 | 0 | 0 | 0 |
| photosymbiotic / semi_infaunal / unattached / mobile | 0 | 0 | 0 | 0 | 0 | 0 | 0 | 0 | 0 | 0 | 0 | 0 | 0 | 0 | 0 | 0 | 0 |
| subsurface_deposit / infaunal_asiphonate / unattached / mobile | 2 | 1 | 3 | 0 | 0 | 0 | 1 | 1 | 2 | 2 | 2 | 3 | 3 | 3 | 3 | 0 | 0 |
| subsurface_deposit / infaunal_siphonate / unattached / immobile | 0 | 0 | 0 | 0 | 0 | 0 | 0 | 0 | 0 | 0 | 0 | 0 | 0 | 0 | 0 | 0 | 0 |
| subsurface_deposit / infaunal_siphonate / unattached / mobile | 2 | 2 | 2 | 0 | 0 | 0 | 4 | 4 | 1 | 2 | 3 | 3 | 3 | 4 | 5 | 1 | 3 |
| surface_deposit / infaunal_siphonate / unattached / mobile | 1 | 1 | 1 | 0 | 2 | 0 | 17 | 16 | 19 | 20 | 21 | 23 | 13 | 13 | 12 | 1 | 2 |
| suspension / borer / byssate / immobile | 0 | 0 | 0 | 0 | 0 | 0 | 3 | 2 | 2 | 3 | 2 | 2 | 2 | 0 | 0 | 0 | 0 |
| suspension / borer / cemented / immobile | 0 | 0 | 0 | 0 | 0 | 0 | 0 | 0 | 0 | 0 | 1 | 1 | 0 | 0 | 0 | 0 | 0 |
| suspension / borer / unattached / immobile | 0 | 0 | 0 | 0 | 0 | 0 | 3 | 4 | 5 | 3 | 5 | 5 | 6 | 6 | 3 | 0 | 0 |
| suspension / borer / unattached / mobile | 0 | 0 | 0 | 0 | 0 | 0 | 2 | 1 | 1 | 2 | 2 | 3 | 2 | 1 | 1 | 0 | 0 |
| suspension / epifaunal / byssate / immobile | 0 | 0 | 0 | 0 | 2 | 0 | 17 | 14 | 14 | 19 | 15 | 18 | 13 | 11 | 11 | 3 | 3 |
| suspension / epifaunal / byssate / mobile | 4 | 4 | 3 | 0 | 2 | 0 | 7 | 7 | 4 | 10 | 7 | 8 | 5 | 6 | 5 | 1 | 1 |
| suspension / epifaunal / byssate / swimming | 2 | 2 | 2 | 0 | 1 | 0 | 4 | 2 | 2 | 3 | 6 | 7 | 4 | 5 | 6 | 1 | 1 |
| suspension / epifaunal / cemented / immobile | 0 | 0 | 0 | 0 | 1 | 0 | 10 | 14 | 13 | 11 | 9 | 12 | 7 | 8 | 7 | 0 | 0 |
| suspension / epifaunal / unattached / immobile | 0 | 0 | 0 | 0 | 0 | 0 | 0 | 0 | 0 | 0 | 0 | 0 | 0 | 0 | 0 | 0 | 0 |
| suspension / epifaunal / unattached / mobile | 0 | 0 | 0 | 0 | 0 | 0 | 0 | 0 | 0 | 0 | 0 | 0 | 0 | 0 | 0 | 0 | 0 |
| suspension / epifaunal / unattached / swimming | 1 | 1 | 1 | 0 | 0 | 0 | 0 | 0 | 0 | 0 | 0 | 0 | 0 | 0 | 0 | 0 | 0 |
| suspension / infaunal_asiphonate / byssate / immobile | 0 | 0 | 0 | 0 | 0 | 0 | 1 | 1 | 1 | 1 | 0 | 0 | 0 | 0 | 0 | 0 | 0 |
| suspension / infaunal_asiphonate / byssate / mobile | 0 | 0 | 0 | 0 | 0 | 0 | 2 | 1 | 1 | 1 | 0 | 0 | 0 | 0 | 0 | 0 | 0 |
| suspension / infaunal_asiphonate / unattached / immobile | 0 | 1 | 0 | 0 | 0 | 0 | 1 | 1 | 1 | 1 | 2 | 2 | 1 | 1 | 1 | 1 | 1 |
| suspension / infaunal_asiphonate / unattached / mobile | 1 | 1 | 0 | 0 | 2 | 0 | 10 | 8 | 10 | 12 | 12 | 10 | 6 | 7 | 5 | 2 | 2 |
| suspension / infaunal_siphonate / byssate / immobile | 0 | 0 | 0 | 0 | 0 | 0 | 3 | 4 | 4 | 4 | 4 | 3 | 2 | 2 | 2 | 0 | 0 |
| suspension / infaunal_siphonate / byssate / mobile | 0 | 0 | 0 | 0 | 0 | 0 | 0 | 0 | 0 | 0 | 0 | 0 | 0 | 0 | 0 | 0 | 0 |
| suspension / infaunal_siphonate / unattached / immobile | 0 | 0 | 0 | 0 | 0 | 0 | 1 | 1 | 1 | 1 | 1 | 2 | 1 | 1 | 1 | 0 | 0 |
| suspension / infaunal_siphonate / unattached / mobile | 3 | 3 | 2 | 0 | 4 | 0 | 50 | 50 | 43 | 58 | 50 | 52 | 40 | 44 | 39 | 3 | 3 |
| suspension / infaunal_siphonate / unattached / swimming | 0 | 0 | 0 | 0 | 0 | 0 | 1 | 1 | 0 | 1 | 1 | 1 | 1 | 1 | 1 | 0 | 0 |
| suspension / nestler / byssate / immobile | 0 | 0 | 0 | 0 | 0 | 0 | 3 | 2 | 1 | 2 | 5 | 5 | 2 | 2 | 1 | 1 | 0 |
| suspension / nestler / byssate / mobile | 0 | 0 | 0 | 0 | 0 | 0 | 0 | 0 | 0 | 0 | 0 | 0 | 0 | 0 | 0 | 0 | 0 |
| suspension / semi_infaunal / byssate / immobile | 0 | 0 | 0 | 0 | 1 | 0 | 2 | 3 | 2 | 3 | 2 | 3 | 1 | 1 | 1 | 0 | 0 |
| suspension / semi_infaunal / unattached / immobile | 0 | 0 | 0 | 0 | 0 | 0 | 0 | 0 | 0 | 0 | 0 | 0 | 0 | 0 | 0 | 0 | 0 |
| suspension / semi_infaunal / unattached / mobile | 1 | 1 | 1 | 0 | 0 | 0 | 1 | 0 | 0 | 0 | 0 | 0 | 0 | 0 | 1 | 0 | 0 |
| suspension / semi_infaunal / unattached / swimming | 0 | 0 | 0 | 0 | 0 | 0 | 1 | 1 | 1 | 1 | 1 | 1 | 1 | 1 | 1 | 0 | 0 |
| **Mean** | **0.5** | **0.5** | **0.5** | **0** | **0.4** | **0** | **3.9** | **3.8** | **3.8** | **4.4** | **4.3** | **4.8** | **3.2** | **3.5** | **3.1** | **0.3** | **0.4** |
| **Max** | **4** | **4** | **3** | **0** | **4** | **0** | **50** | **50** | **43** | **58** | **50** | **52** | **40** | **44** | **39** | **3** | **3** |

| **West Pacific** | **-80** | **-70** | **-60** | **-50** | **-40** | **-30** | **-20** | **-10** | **0** | **10** | **20** | **30** | **40** | **50** | **60** | **70** | **80** |
| --- | --- | --- | --- | --- | --- | --- | --- | --- | --- | --- | --- | --- | --- | --- | --- | --- | --- |
| carnivore / epifaunal / byssate / mobile | 1 | 0 | 0 | 1 | 2 | 2 | 0 | 3 | 2 | 3 | 3 | 2 | 1 | 2 | 0 | 1 | 0 |
| carnivore / epifaunal / byssate / swimming | 0 | 0 | 0 | 0 | 0 | 0 | 0 | 1 | 1 | 1 | 1 | 1 | 0 | 0 | 0 | 0 | 0 |
| carnivore / infaunal_siphonate / unattached / mobile | 2 | 2 | 0 | 3 | 11 | 4 | 6 | 4 | 10 | 14 | 11 | 19 | 7 | 1 | 0 | 2 | 0 |
| chemosymbiotic / infaunal_asiphonate / unattached / mobile | 0 | 0 | 0 | 0 | 1 | 0 | 0 | 0 | 1 | 1 | 1 | 1 | 1 | 1 | 0 | 0 | 0 |
| chemosymbiotic / infaunal_siphonate / unattached / mobile | 2 | 3 | 0 | 11 | 20 | 14 | 19 | 14 | 12 | 21 | 13 | 22 | 10 | 3 | 2 | 3 | 0 |
| chemosymbiotic / infaunal_siphonate / unattached / swimming | 0 | 0 | 0 | 0 | 0 | 1 | 0 | 0 | 0 | 0 | 0 | 1 | 1 | 0 | 0 | 0 | 0 |
| photosymbiotic / epifaunal / byssate / immobile | 0 | 0 | 0 | 0 | 0 | 2 | 3 | 3 | 3 | 3 | 3 | 0 | 0 | 0 | 0 | 0 | 0 |
| photosymbiotic / epifaunal / unattached / mobile | 0 | 0 | 0 | 0 | 0 | 1 | 1 | 1 | 1 | 1 | 1 | 1 | 0 | 0 | 0 | 0 | 0 |
| photosymbiotic / semi_infaunal / unattached / mobile | 0 | 0 | 0 | 0 | 0 | 1 | 2 | 2 | 2 | 2 | 2 | 1 | 0 | 0 | 0 | 0 | 0 |
| subsurface_deposit / infaunal_asiphonate / unattached / mobile | 2 | 2 | 0 | 3 | 4 | 2 | 2 | 1 | 3 | 3 | 5 | 5 | 5 | 2 | 2 | 1 | 0 |
| subsurface_deposit / infaunal_siphonate / unattached / immobile | 0 | 0 | 0 | 0 | 0 | 0 | 0 | 0 | 0 | 0 | 0 | 0 | 0 | 0 | 0 | 0 | 0 |
| subsurface_deposit / infaunal_siphonate / unattached / mobile | 2 | 2 | 0 | 0 | 2 | 5 | 6 | 7 | 9 | 8 | 8 | 13 | 7 | 5 | 3 | 3 | 0 |
| surface_deposit / infaunal_siphonate / unattached / mobile | 1 | 1 | 0 | 4 | 23 | 44 | 45 | 29 | 32 | 58 | 45 | 42 | 20 | 5 | 3 | 2 | 0 |
| suspension / borer / byssate / immobile | 0 | 0 | 0 | 0 | 2 | 4 | 3 | 6 | 4 | 6 | 6 | 6 | 2 | 1 | 0 | 0 | 0 |
| suspension / borer / cemented / immobile | 0 | 0 | 0 | 0 | 1 | 0 | 0 | 0 | 0 | 1 | 2 | 2 | 0 | 0 | 0 | 0 | 0 |
| suspension / borer / unattached / immobile | 0 | 0 | 0 | 3 | 5 | 6 | 6 | 7 | 13 | 16 | 15 | 13 | 13 | 2 | 1 | 0 | 0 |
| suspension / borer / unattached / mobile | 0 | 0 | 0 | 0 | 3 | 1 | 1 | 1 | 2 | 3 | 3 | 5 | 1 | 0 | 0 | 0 | 0 |
| suspension / epifaunal / byssate / immobile | 0 | 0 | 0 | 0 | 25 | 24 | 27 | 20 | 23 | 38 | 38 | 38 | 22 | 7 | 5 | 2 | 0 |
| suspension / epifaunal / byssate / mobile | 4 | 4 | 0 | 2 | 13 | 12 | 13 | 16 | 14 | 18 | 19 | 20 | 4 | 1 | 1 | 1 | 0 |
| suspension / epifaunal / byssate / swimming | 2 | 2 | 0 | 0 | 5 | 9 | 8 | 12 | 13 | 17 | 12 | 16 | 8 | 1 | 0 | 0 | 0 |
| suspension / epifaunal / cemented / immobile | 0 | 0 | 0 | 6 | 16 | 14 | 17 | 11 | 22 | 24 | 23 | 18 | 15 | 1 | 1 | 0 | 0 |
| suspension / epifaunal / unattached / immobile | 0 | 0 | 0 | 0 | 0 | 2 | 2 | 2 | 2 | 2 | 2 | 0 | 0 | 0 | 0 | 0 | 0 |
| suspension / epifaunal / unattached / mobile | 0 | 0 | 0 | 1 | 2 | 1 | 0 | 0 | 0 | 1 | 0 | 0 | 0 | 0 | 0 | 0 | 0 |
| suspension / epifaunal / unattached / swimming | 1 | 1 | 0 | 0 | 1 | 1 | 1 | 2 | 2 | 2 | 1 | 1 | 0 | 0 | 0 | 0 | 0 |
| suspension / infaunal_asiphonate / byssate / immobile | 0 | 0 | 0 | 1 | 3 | 2 | 1 | 0 | 0 | 3 | 3 | 3 | 2 | 1 | 0 | 0 | 0 |
| suspension / infaunal_asiphonate / byssate / mobile | 0 | 0 | 0 | 0 | 0 | 0 | 1 | 0 | 2 | 5 | 2 | 5 | 3 | 1 | 0 | 0 | 0 |
| suspension / infaunal_asiphonate / unattached / immobile | 0 | 0 | 0 | 0 | 3 | 7 | 6 | 5 | 8 | 10 | 10 | 6 | 4 | 0 | 0 | 1 | 0 |
| suspension / infaunal_asiphonate / unattached / mobile | 1 | 1 | 0 | 13 | 27 | 25 | 17 | 12 | 12 | 23 | 23 | 22 | 14 | 5 | 3 | 2 | 0 |
| suspension / infaunal_siphonate / byssate / immobile | 0 | 0 | 0 | 1 | 2 | 4 | 3 | 3 | 8 | 10 | 7 | 7 | 4 | 0 | 0 | 0 | 0 |
| suspension / infaunal_siphonate / byssate / mobile | 0 | 0 | 0 | 0 | 2 | 0 | 0 | 0 | 0 | 1 | 1 | 1 | 0 | 0 | 0 | 0 | 0 |
| suspension / infaunal_siphonate / unattached / immobile | 0 | 0 | 0 | 1 | 2 | 3 | 3 | 3 | 1 | 5 | 3 | 5 | 4 | 0 | 0 | 0 | 0 |
| suspension / infaunal_siphonate / unattached / mobile | 3 | 3 | 0 | 21 | 84 | 90 | 89 | 85 | 90 | 145 | 121 | 136 | 83 | 19 | 9 | 8 | 0 |
| suspension / infaunal_siphonate / unattached / swimming | 0 | 0 | 0 | 0 | 0 | 1 | 1 | 1 | 1 | 1 | 1 | 1 | 0 | 0 | 0 | 0 | 0 |
| suspension / nestler / byssate / immobile | 0 | 0 | 0 | 2 | 4 | 6 | 8 | 5 | 5 | 9 | 9 | 9 | 5 | 1 | 0 | 1 | 0 |
| suspension / nestler / byssate / mobile | 0 | 0 | 0 | 0 | 0 | 3 | 3 | 3 | 4 | 4 | 4 | 2 | 1 | 0 | 0 | 0 | 0 |
| suspension / semi_infaunal / byssate / immobile | 0 | 0 | 0 | 0 | 4 | 5 | 5 | 5 | 3 | 5 | 4 | 4 | 1 | 0 | 0 | 0 | 0 |
| suspension / semi_infaunal / unattached / immobile | 0 | 0 | 0 | 0 | 0 | 0 | 0 | 0 | 0 | 0 | 0 | 0 | 1 | 1 | 0 | 0 | 0 |
| suspension / semi_infaunal / unattached / mobile | 1 | 1 | 0 | 0 | 2 | 0 | 1 | 0 | 1 | 2 | 4 | 5 | 4 | 1 | 1 | 0 | 0 |
| suspension / semi_infaunal / unattached / swimming | 0 | 0 | 0 | 0 | 1 | 1 | 0 | 0 | 0 | 1 | 1 | 1 | 1 | 0 | 0 | 0 | 0 |
| **Mean** | **0.5** | **0.5** | **0** | **1.8** | **6.9** | **7.6** | **7.6** | **6.7** | **7.8** | **11.9** | **10.4** | **11.1** | **6.2** | **1.5** | **0.7** | **0.6** | **0** |
| **Max** | **4** | **4** | **0** | **21** | **84** | **90** | **89** | **85** | **90** | **145** | **121** | **136** | **83** | **19** | **9** | **8** | **0** |

| **East Pacific** | **-80** | **-70** | **-60** | **-50** | **-40** | **-30** | **-20** | **-10** | **0** | **10** | **20** | **30** | **40** | **50** | **60** | **70** | **80** |
| --- | --- | --- | --- | --- | --- | --- | --- | --- | --- | --- | --- | --- | --- | --- | --- | --- | --- |
| carnivore / epifaunal / byssate / mobile | 1 | 0 | 0 | 0 | 0 | 0 | 1 | 1 | 1 | 1 | 2 | 2 | 2 | 3 | 2 | 1 | 0 |
| carnivore / epifaunal / byssate / swimming | 0 | 0 | 0 | 0 | 0 | 0 | 0 | 0 | 0 | 0 | 0 | 0 | 0 | 0 | 0 | 0 | 0 |
| carnivore / infaunal_siphonate / unattached / mobile | 2 | 2 | 1 | 0 | 0 | 0 | 1 | 4 | 6 | 0 | 5 | 8 | 5 | 3 | 2 | 1 | 0 |
| chemosymbiotic / infaunal_asiphonate / unattached / mobile | 0 | 0 | 0 | 0 | 0 | 0 | 0 | 0 | 0 | 0 | 2 | 1 | 1 | 0 | 0 | 0 | 0 |
| chemosymbiotic / infaunal_siphonate / unattached / mobile | 2 | 3 | 4 | 4 | 0 | 0 | 1 | 10 | 8 | 7 | 18 | 14 | 3 | 8 | 2 | 3 | 0 |
| chemosymbiotic / infaunal_siphonate / unattached / swimming | 0 | 0 | 0 | 0 | 0 | 0 | 0 | 0 | 0 | 1 | 1 | 1 | 0 | 1 | 0 | 0 | 0 |
| photosymbiotic / epifaunal / byssate / immobile | 0 | 0 | 0 | 0 | 0 | 0 | 0 | 0 | 0 | 0 | 0 | 0 | 0 | 0 | 0 | 0 | 0 |
| photosymbiotic / epifaunal / unattached / mobile | 0 | 0 | 0 | 0 | 0 | 0 | 0 | 0 | 0 | 0 | 0 | 0 | 0 | 0 | 0 | 0 | 0 |
| photosymbiotic / semi_infaunal / unattached / mobile | 0 | 0 | 0 | 0 | 0 | 0 | 0 | 0 | 0 | 0 | 0 | 0 | 0 | 0 | 0 | 0 | 0 |
| subsurface_deposit / infaunal_asiphonate / unattached / mobile | 2 | 2 | 3 | 3 | 2 | 3 | 2 | 3 | 3 | 2 | 4 | 4 | 3 | 3 | 2 | 1 | 0 |
| subsurface_deposit / infaunal_siphonate / unattached / immobile | 0 | 0 | 0 | 0 | 0 | 0 | 0 | 0 | 0 | 0 | 0 | 0 | 0 | 0 | 0 | 0 | 0 |
| subsurface_deposit / infaunal_siphonate / unattached / mobile | 2 | 2 | 4 | 3 | 1 | 0 | 0 | 3 | 3 | 3 | 5 | 8 | 6 | 6 | 6 | 4 | 0 |
| surface_deposit / infaunal_siphonate / unattached / mobile | 1 | 1 | 1 | 2 | 3 | 2 | 3 | 27 | 36 | 27 | 34 | 28 | 13 | 13 | 7 | 3 | 0 |
| suspension / borer / byssate / immobile | 0 | 0 | 0 | 1 | 0 | 1 | 2 | 7 | 8 | 5 | 6 | 5 | 1 | 1 | 0 | 0 | 0 |
| suspension / borer / cemented / immobile | 0 | 0 | 0 | 0 | 0 | 0 | 0 | 0 | 0 | 0 | 0 | 0 | 0 | 0 | 0 | 0 | 0 |
| suspension / borer / unattached / immobile | 0 | 0 | 4 | 2 | 4 | 3 | 3 | 12 | 13 | 10 | 15 | 10 | 3 | 2 | 3 | 0 | 0 |
| suspension / borer / unattached / mobile | 0 | 0 | 0 | 1 | 1 | 2 | 2 | 3 | 5 | 5 | 5 | 3 | 2 | 2 | 0 | 0 | 0 |
| suspension / epifaunal / byssate / immobile | 0 | 0 | 2 | 4 | 7 | 6 | 6 | 16 | 15 | 16 | 19 | 16 | 6 | 8 | 6 | 5 | 0 |
| suspension / epifaunal / byssate / mobile | 4 | 4 | 3 | 5 | 5 | 1 | 1 | 10 | 8 | 7 | 9 | 8 | 2 | 2 | 2 | 1 | 0 |
| suspension / epifaunal / byssate / swimming | 2 | 2 | 2 | 1 | 1 | 1 | 1 | 2 | 3 | 3 | 3 | 3 | 2 | 2 | 2 | 1 | 0 |
| suspension / epifaunal / cemented / immobile | 0 | 0 | 1 | 0 | 2 | 1 | 1 | 15 | 16 | 16 | 20 | 13 | 4 | 4 | 2 | 1 | 0 |
| suspension / epifaunal / unattached / immobile | 0 | 0 | 0 | 0 | 1 | 1 | 1 | 1 | 1 | 1 | 1 | 1 | 0 | 0 | 0 | 0 | 0 |
| suspension / epifaunal / unattached / mobile | 0 | 0 | 0 | 0 | 0 | 0 | 0 | 3 | 3 | 3 | 3 | 2 | 0 | 0 | 0 | 0 | 0 |
| suspension / epifaunal / unattached / swimming | 1 | 1 | 0 | 0 | 0 | 0 | 0 | 0 | 0 | 0 | 0 | 0 | 0 | 0 | 0 | 0 | 0 |
| suspension / infaunal_asiphonate / byssate / immobile | 0 | 0 | 0 | 0 | 0 | 0 | 0 | 0 | 1 | 1 | 1 | 1 | 0 | 1 | 1 | 0 | 0 |
| suspension / infaunal_asiphonate / byssate / mobile | 0 | 0 | 0 | 0 | 1 | 0 | 0 | 3 | 4 | 4 | 4 | 1 | 0 | 2 | 0 | 0 | 0 |
| suspension / infaunal_asiphonate / unattached / immobile | 0 | 0 | 0 | 0 | 0 | 0 | 0 | 6 | 6 | 6 | 6 | 1 | 0 | 0 | 0 | 1 | 0 |
| suspension / infaunal_asiphonate / unattached / mobile | 1 | 1 | 2 | 2 | 2 | 1 | 2 | 13 | 13 | 12 | 14 | 9 | 5 | 6 | 6 | 4 | 0 |
| suspension / infaunal_siphonate / byssate / immobile | 0 | 0 | 0 | 0 | 0 | 0 | 2 | 8 | 8 | 6 | 6 | 5 | 1 | 1 | 0 | 0 | 0 |
| suspension / infaunal_siphonate / byssate / mobile | 0 | 0 | 0 | 0 | 0 | 0 | 0 | 0 | 0 | 0 | 0 | 0 | 0 | 0 | 0 | 0 | 0 |
| suspension / infaunal_siphonate / unattached / immobile | 0 | 0 | 1 | 0 | 0 | 0 | 0 | 0 | 0 | 0 | 0 | 2 | 2 | 2 | 1 | 1 | 0 |
| suspension / infaunal_siphonate / unattached / mobile | 3 | 3 | 9 | 13 | 20 | 18 | 17 | 64 | 85 | 62 | 93 | 66 | 33 | 33 | 28 | 11 | 0 |
| suspension / infaunal_siphonate / unattached / swimming | 0 | 0 | 0 | 0 | 0 | 0 | 0 | 1 | 1 | 1 | 1 | 1 | 0 | 0 | 0 | 0 | 0 |
| suspension / nestler / byssate / immobile | 0 | 0 | 1 | 1 | 2 | 1 | 1 | 5 | 5 | 5 | 5 | 5 | 4 | 2 | 1 | 1 | 0 |
| suspension / nestler / byssate / mobile | 0 | 0 | 0 | 0 | 0 | 0 | 0 | 0 | 1 | 1 | 1 | 1 | 0 | 0 | 0 | 0 | 0 |
| suspension / semi_infaunal / byssate / immobile | 0 | 0 | 0 | 0 | 0 | 0 | 0 | 2 | 3 | 2 | 2 | 2 | 0 | 0 | 0 | 0 | 0 |
| suspension / semi_infaunal / unattached / immobile | 0 | 0 | 0 | 0 | 0 | 0 | 0 | 0 | 0 | 0 | 0 | 1 | 1 | 1 | 1 | 0 | 0 |
| suspension / semi_infaunal / unattached / mobile | 1 | 1 | 0 | 1 | 1 | 1 | 0 | 3 | 4 | 3 | 3 | 4 | 2 | 2 | 2 | 1 | 0 |
| suspension / semi_infaunal / unattached / swimming | 0 | 0 | 0 | 0 | 0 | 0 | 0 | 0 | 0 | 0 | 0 | 0 | 0 | 0 | 0 | 0 | 0 |
| **Mean** | **0.5** | **0.5** | **0.9** | **1.1** | **1.3** | **1.0** | **1.2** | **5.6** | **6.6** | **5.3** | **7.3** | **5.7** | **2.5** | **2.7** | **1.9** | **1.0** | **0** |
| **Max** | **4** | **4** | **9** | **13** | **20** | **18** | **17** | **64** | **85** | **62** | **93** | **66** | **33** | **33** | **28** | **11** | **0** |

The mean and maximum number of genera per functional group decreases from low to high latitude (see legend of Figure S8). Body size was not included in these functional groups.
