# Supplementary material for: Origination and Immigration Drive Latitudinal Gradients in Marine Functional Diversity
Source: PLoS One. 2014 Jul 18;9(7):e101494. doi: 10.1371/journal.pone.0101494 (PMC4103801; doi:10.1371/journal.pone.0101494)
Supplement: Text S1 — Supplementary methods. (PDF) [file pone.0101494.s009.pdf]

Supporting Methods

Supporting Methods

Functional Divergence: Functional divergence (FDiv) measures the degree to which taxa are spread out in trait space. In a PCoA ordination, FDiv is quantified as the mean distance from each taxon to the centroid. This is a simplification of Villeger et al's method [1] in that it considers all taxa in calculating the centroid, and not just taxa forming the vertices of the convex hull. All calculations and null models were performed as described above for FEve. Observed FDiv exhibits hemispheric asymmetry at the poles — although the observations at both poles are within null expectations (Figure S7), arctic points are at the low end of that expectation while Antarctic points are at the high end. We hypothesized that this asymmetry could be the result of different extinction histories at the poles [2], and asked if adding extinct polar genera back into the dataset would resolve this asymmetry. Adding extinct Arctic genera partially resolves the hemispheric asymmetry for the Pacific basin, but has the opposite effect or no effect in the West and East Atlantic, respectively.

1. Villeger S, Mason NWH, Mouillot D (2008) New multidimensional functional diversity indices for a multifaceted framework in functional ecology. Ecology 89: 2290-2301.
2. Krug AZ, Jablonski D, Roy K, Beu AG (2010) Differential extinction and the contrasting structure of polar marine faunas. PLoS ONE 5: e15362-e15362.
